# Morphology and Structure of Brass–Invar Weld Interface after Explosive Welding

**DOI:** 10.3390/ma15238587

**Published:** 2022-12-01

**Authors:** Andrey Malakhov, Alexander Epishin, Igor Denisov, Ivan Saikov, Gert Nolze

**Affiliations:** 1 Merzhanov Institute of Structural Macrokinetics and Materials Science of Russian Academy of Sciences, 142432 Chernogolovka, Russia; 2 Federal Institute for Materials Research and Testing, 12205 Berlin, Germany

**Keywords:** explosive welding, thermobimetal, grain structure, brass, Invar

## Abstract

This paper presents the results of a study of the morphology and structure at the weld interface in a brass–Invar bimetal, which belongs to the class of so-called thermostatic bimetals, or thermobimetals. The structure of the brass–Invar weld interface was analyzed using optical microscopy and scanning electron microscopy (SEM), with the use of energy-dispersive X-ray (EDX) spectrometry and back-scattered electron diffraction (BSE) to identify the phases. The distribution of the crystallographic orientation of the grains at the weld interface was obtained using an e-Flash HR electron back-scatter diffraction (EBSD) detector and a forward-scatter detector (FSD). The results of the study indicated that the weld interface had the wavy structure typical of explosive welding. The wave crests and troughs showed the presence of melted zones consisting of a disordered Cu–Zn–Fe–Ni solid solution and undissolved Invar particles. The pattern quality map showed that the structure of brass and Invar after explosive welding consisted of grains that were strongly elongated towards the area of the highest intensive plastic flow. In addition, numerous deformation twins, dislocation accumulations and shear bands were observed. Thus, based on the results of this study, the mechanism of Cu–Zn–Fe–Ni structure formation can be proposed.

## 1. Introduction

The technological development of many fields of mechanical engineering depends on the progress in materials science. Without materials capable of carrying the corresponding load, even the most advanced design is meaningless. Therefore, the designing of new constructions is always constrained by the limited properties of available materials. In this regard, bimetals are the most promising materials. Bimetals are materials consisting of two or more metallic layers, which makes them more flexible and suitable for a broad range of applications.

A particular type of bimetals is thermostatic bimetals (thermobimetals). Thermobimetals are bimetals consisting of metallic layers with different coefficients of thermal expansion (CTEs) [1,2]. The layer with the highest CTE is called active, while the other layer is passive. These layers are commonly made using Invar (a Ni–Fe alloy) and brass (a Cu–Zn alloy), with Invar being the passive layer and brass being the active layer. Upon heating, the active layer of the bimetal is subjected compressive stress, while the passive layer is subjected to expansion stress. As a result, the bimetal is bent towards the passive layer. This property has allowed thermobimetals to be used as thermal actuators in a large number of devices.

Thermobimetals are most commonly manufactured in the form of bimetallic strips through the process of cold roll bonding. Other methods of manufacturing include casting and electron beam deposition. Cold roll bonding is an effective but labor-intensive process requiring heat treatment after each stage of rolling. Casting and electron beam deposition, on the other hand, change the thicknesses of the initial metals. In addition, casting and electron beam deposition heat and melt the initial metals, which leads to the formation of brittle intermetallic compounds in the weld joint. This reduces the mechanical properties of the thermobimetal.

Due to the above-mentioned limits, the industry of thermobimetal production has been seeking ways to increase the weld strength while reducing electrical energy consumption. Explosive welding (EW) has great potential to address both issues. This welding method is possible due to the high pressure produced by the explosion [3,4] and has several advantages over the above-mentioned thermobimetal production methods [5].

The explosive nature of this welding technique, however, poses a number of challenges. One of them is the fact that the shock waves produced by the detonation of the explosives obstruct the direct monitoring of the welding process. The high pressure, on the other hand, produces such high mechanical stress within the metals that it is almost impossible to analyze their properties during the welding process. As a result, researchers have to make certain assumptions when studying the mechanisms of the weld formation. Consequently, most studies in this field focus on the structure and properties of the weld interface after its formation.

EW is considered a solid-state process. Nevertheless, the resulting considerable plastic deformation and the impact of the shock-compressed gas liberated in the gap between the metals often produce melted zones [6,7]. As these zones form, structural changes occur at the weld interface, which can produce both metastable non-equilibrium phases and amorphous structures. Notably, when this happens in the welding of similar metals (e.g., Cu + Cu), the strength of the weld is hardly affected. The welding of dissimilar metals (e.g., Cu + Ti), however, produces brittle intermetallic compounds, solid solutions or complex eutectic phases at the weld interface, which in most cases considerably reduces the strength of the weld. Furthermore, the difference in the properties of dissimilar metals can produce zones of fine-grained microstructure with high microhardness and zones of recrystallized grains [8,9]. All of this leads to an inhomogeneous structure of the weld interface and reduces its strength.

Notably, few articles on the EW of brass with Invar have ever been published. Only two works, by R.V. Tamhankar and J. Ramesam, can be noted, which were written in the mid-1970s. These works describe the intermediate zones, areas of severe plastic flow, extensive twinning and narrow bands of recrystallized grains located at the weld interface. However, the results of these studies did not demonstrate any definite mechanism of the structure formation apart from suggesting the elemental mixture composition of the intermediate zones.

This paper reports the results obtained when energy-dispersive X-ray (EDX) spectrometry and electron back-scatter diffraction (EBSD) were applied to analyze the microstructure of the brass (CuZn37)-Invar 36 weld interface and to evaluate the effect of the severe plastic deformation on the shape and size of the grains of the initial materials. These and earlier results [10,11] enabled us to propose the mechanism of the formation of melted zones at the weld interface. Thus, the paper presents a more detailed understanding of the nature of the structural transformations at the brass–Invar weld interface, which could help to produce stronger thermobimetals.

## 2. Materials and Methods

**Figure 1:**
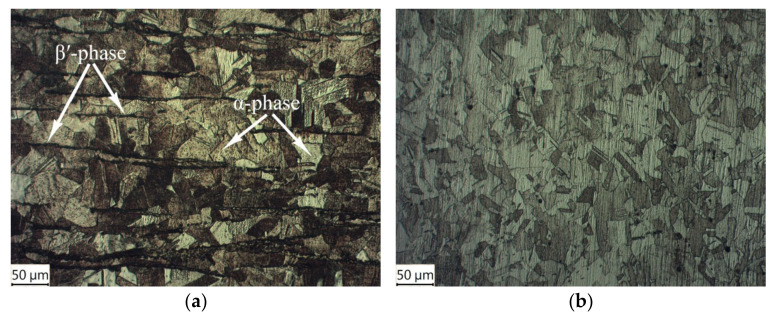
Structure of the initial plates: (**a**) brass CuZn37; (**b**) Invar 36.

In the experiment, commercially available plates of Invar 36 and brass (CuZn37) were used. Initially, the plates had the dimensions of 8 × 200 × 300 mm (Invar 36 plate) and 12 × 200 × 500 mm (brass plate). CuZn37 is single-phase brass that has α-solid solution (Zn in Cu) structure with a face-centered cubic unit cell (fcc). However, depending on the cooling conditions during the production process, some traces of the β′-phase with body-centered cubic unit cell (bcc) are possible (Figure 1a). Invar is also a single-phase alloy; its structure is represented by a γ-solid solution (fcc) of Ni in Fe (see Figure 1b).

The explosive used was a mixture of microporous ammonium nitrate and diesel oil (ANFO). The detonation velocity was 3000–3200 m/s. The distance between the flying and static plates was about 6 mm. For other details of the EW experiments, see [11].

The structure of the bimetals was investigated using optical microscopy and scanning electron microscopy (SEM), with the use of energy-dispersive X-ray (EDX) spectrometry and back-scattered electron diffraction (BSE) to identify phases. An optical analysis was performed with a Lomo METAM LB-34 microscope. Two microscopes were used in the SEM investigations: a Leo GEMINI 1530 SEM with a Bruker EDS XFlash 5030 EDX spectrometer, an e-Flash HR electron back-scatter diffraction (EBSD) detector and a forward-scatter detector (FSD); and a Zeiss Ultraplus SEM with an Oxford INCA 350 EDX spectrometer. To study the weld interface structure, the bimetallic plates were cut perpendicular to the weld interface and then ground on abrasive papers and polished with diamond pastes with a particle size less than 1 μm. The grain structure of the alloys was revealed by etching with a 1:3 mixtures of HNO_3_ and HCl for Invar and concentrated HNO_3_ for brass. For EBSD, the samples were additionally polished using a suspension of colloidal silica with a particle size of 25 nm.

Vickers microhardness (HV) was measured using a microhardness tester (PMT-3). Loads of 50 and 100 g were applied for 10 and 15 s.

## 3. Results

**Figure 2:**
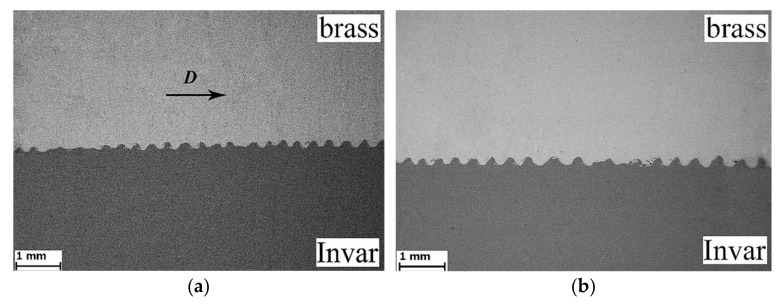
SEM images of the weld interface of brass (CuZn37) and Invar 36 after EW: (**a**) initial part; (**b**) final part; arrow D shows the detonation direction.

Figure 2 shows the wavy structure of the weld interface between brass (CuZn37) and Invar 36 after EW. The wave amplitude and wavelength in the initial part of the bimetal were approximately 140 and 310 μm, respectively (Figure 2a); in the final part of the bimetal, they were 300 and 500 μm, respectively (Figure 2b). It is well known that the composite plates obtained using EW are usually joined together in a flat or wavy interface. The flat interface turned into the wavy interface as the explosive thickness or stand-off distance between the initial plates increased. The shape, period and amplitude of the waves are functions of the particular metal combination, the thickness of the flyer plate, and the collision conditions experienced. In addition, a flat weld interface is indicative of a weld made using the lower limits of collision energy; a wavy interface is indicative of the use of the upper limits.

The formation of the wavy structure at the weld interface has attracted a lot of attention among researchers [12,13,14,15]. At present, the hydrodynamic theory is the main theory that describes the dynamic behavior of a material under high-velocity oblique collision; therefore, it is most often used to explain wave formation [16,17]. For example, in [18], smoothed-particle hydrodynamics (SPH) was used to analyze high-velocity oblique collision. As a result of the numerical simulation and experimental studies, the wave formation mechanism was described. In addition, an atomistic simulation [19] and a molecular dynamics approach [20] were used for a more detailed understanding of wave formation at the weld interface. An increase in the wave amplitude and wavelength in the final part of the bimetal occur due to the impact of shock-compressed gas on the welded surfaces and the considerable plastic deformation of brass [11].

**Figure 3:**
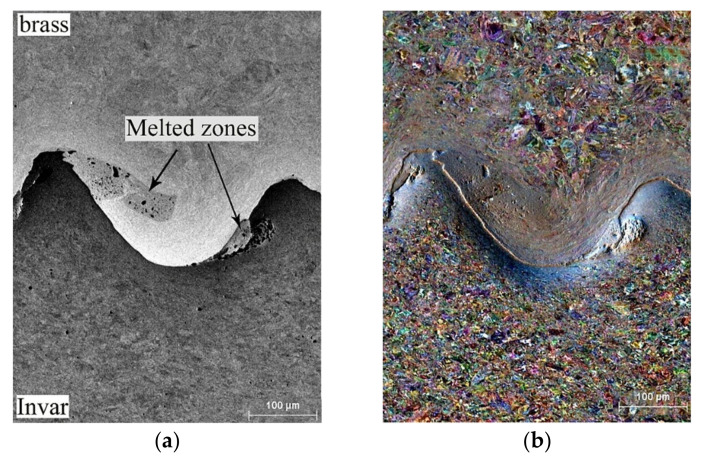
BSE (**a**) and FSD (**b**) images of the weld interface.

The metallographic analysis of the weld interface revealed melted zones located at the wave crests and troughs (Figure 3a) and zones of severe plastic deformation of grains (Figure 3b). These melted zones had the spongy structure, which indicated a significant amount of gases and nonmetallic particles.

Directly at the weld interface, one could see the curved traces of located plastic flow indicating the high-rate severe plastic deformation of the metal during EW (Figure 3b). Such severe plastic deformation of the grains resulted in a strongly deformed grain structure with significant lattice distortions and residual stresses. The deformation of the brass grains was more pronounced and extended to a greater depth than that of Invar grains. The depth of the deformation of brass grains was about 100–150 μm, while that of Invar grains was about 50–70 μm.

The results of the microhardness measurements at the weld interface [11] showed that the microhardness of brass in the strain hardening zone was about 230 HV, which was 35% higher than its microhardness in the initial state. The microhardness of Invar was 270 HV, which was 19% higher than that in the initial state. Thus, the degree of brass strain hardening exceeded the strain hardening degree of Invar by about two times. This effect was obviously caused by an increase in dislocation density and the formation of a cellular dislocation substructure clogging the dislocation movement. The obtained data on microhardness also indicated a grater reduction in brass ductility as compared with Invar.

**Figure 4:**
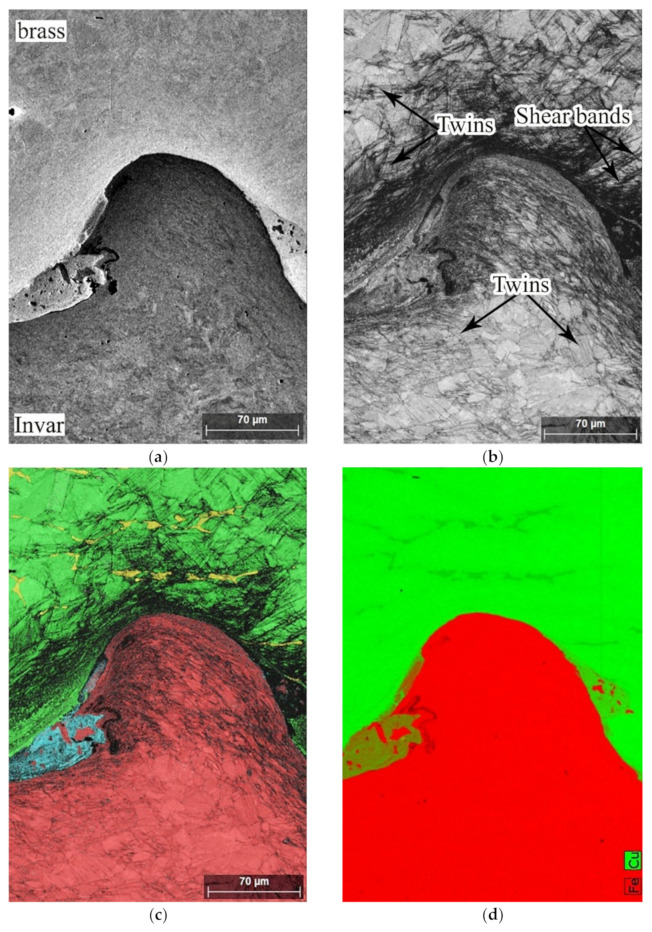
Structure and phase composition of the materials near the interface: (**a**–**c**) BSD, pattern quality and phase distribution map, respectively. In (**c**), brass (CuZn) is green; Invar (FeNi) is red; the melted zone (Fe–Ni–Cu–Zn) is cyan; and the β′-phase (CuZn) is yellow. (**d**) Element distribution map of Fe + Cu.

Figure 4a shows the structure of a single wave at the weld interface. The pattern quality map (Figure 4b) showed that the structure of brass and Invar after EW consisted of grains that were severely outstretched towards the highest intensive plastic flow. In addition, there were numerous deformation twins, and shear bands were revealed. The shear bands were usually localized in the direction aligned parallel to the maximum tangential stresses.

The phase distribution map (Figure 4c) and the element distribution maps (count maps) for Fe, Cu (Figure 4d) revealed the presence at the weld interface of the Cu–Zn–Fe–Ni structure with unmelted Invar particles. These Invar particles were formed as a result of fragmentation by way of the granulating of the structure [21]. The fragmentation of a structure is detailed in the study of [22].

**Figure 5:**
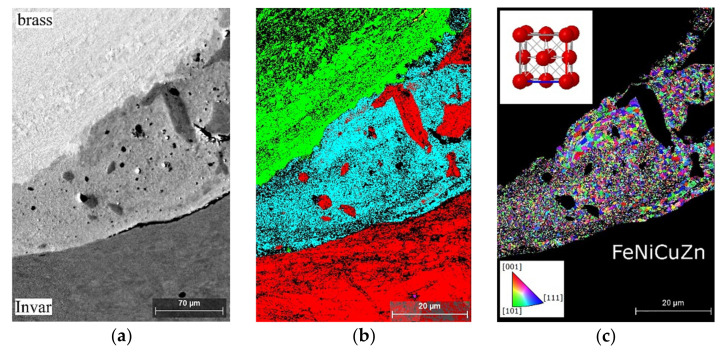
BSD images of brass–Invar weld interface and inverse pole figure (IPF-X) map for Cu–Zn–Fe–Ni: (**a**) image of porous structure of the melted zone; (**b**) phase distribution map of the weld interface, where brass (CuZn) is green, Invar (FeNi) is red and the melted zone (Fe–Ni–Cu–Zn) is cyan; (**c**) IPF-X map of the melted zone.

Figure 5 shows the porous structure of the melted zone (Figure 5a), the phase composition and particles of unmelted Invar (Figure 5b), and the finely dispersed heterogeneous grain structure of Cu–Zn–Fe–Ni (Figure 5c). The Invar particles ranged in size from 0.1 µm (Figure 5b) to 20 µm (Figure 4c). Figure 5c shows the area of mixed composition Cu–Zn–Fe–Ni located at the weld interface. The average grain size in this area was 200 nm, which was significantly smaller than that of brass and Invar. This is because this area was first melted during EW and after that rapidly cooled. This process is detailed and described in the work of [18]. In addition, Figure 5c shows that the shape of the Cu–Zn–Fe–Ni grains was isotropic; consequently, these grains were not plastically deformed. This indicated that the Cu–Zn–Fe–Ni structure was in the liquid state during the plastic deformation of the weld interface. According to the Cu–Zn–Fe ternary phase diagram, the Cu–Zn–Fe–Ni structure had the following phase composition: γ-solid solution (fcc) Cu in Zn + α-Fe (bcc). Despite this, the EBSD maps did not show α-Fe (bcc) in the Cu–Zn–Fe–Ni structure. This was probably due to the presence of Ni, which is known to be the fcc stabilizing element for Fe [23]. Thus, it can be expected that the Cu–Zn–Fe–Ni structure was a disordered fcc solid solution.

**Figure 6:**
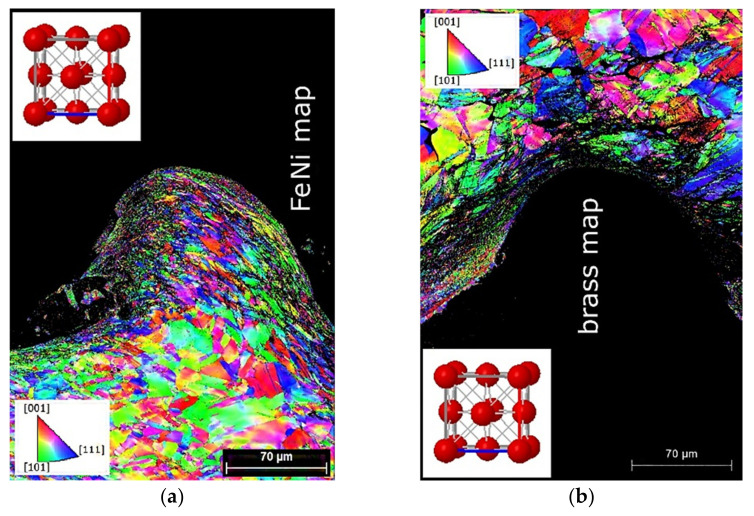
IPF-X maps of the grain structure near the weld interface: (**a**) Invar; (**b**) brass. The reference X-axis is directed vertically upwards. The color coding of the orientation is given by STT.

The crystals had different orientations or three-dimensional configurations relative to the reference frame. Figure 6 shows IPF-X maps characterizing the distribution of the crystallographic orientations. In the IPF-X maps, each individual orientation of crystals is colored differently, and the color coding of the orientations is presented in Standard Stereographic Triangle (SST), shown as an inset in the corner of the IPF image. Figure 6 reveals both the primary grain boundaries and the strongly deformed grain structure of brass and Invar developed during plastic deformation. In addition, we observed preferentially formed recrystallized grains in the vicinity of and along the weld interface. The different colors of the deformed Invar (Figure 6a) and brass (Figure 6b) grains indicate the absence of the preferred lattice orientation. In addition, Figure 6b clearly shows a significant number of dislocations located in brass.

**Figure 7:**
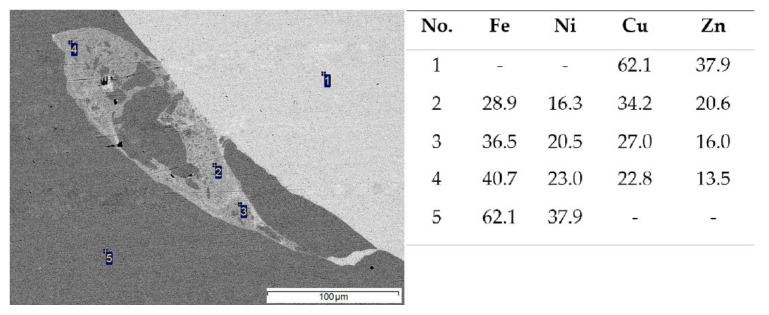
SEM image of a melted zone and the results of point EDS analyses (wt %).

From the results of the performed EDS analyses (Figure 7), it followed that the distribution of elements in the Cu–Zn–Fe–Ni structure was almost uniform. This indicated a complete mutual mixing of brass and Invar, which can occur by diffusion in the liquid phase formed during EW. As can be seen from the results of the point EDS analyses, Fe and Ni were present in lower amounts than Invar. Similarly, Cu and Zn were also depleted, though to a much smaller extent.

Thus, based on the results of this study, the mechanism of Cu–Zn–Fe–Ni structure formation can be proposed. It consists of the following states: First, the process of EW heats the materials at the weld interface due to the effect of the shock-compressed gas and significant plastic deformation. Apparently, brass melts first (T_m.p._ = 906 °C) and has a much lower melting point than Invar (T_m.p._ = 1425 °C). Then, Invar dissolves in the melted brass. Fe and Ni quickly diffuse from Invar into the liquid phase of brass, since the diffusion mobility of the liquid-phase atoms is several orders of magnitude higher than that of atoms in the solid phase. Thus, Fe and Ni are evenly distributed in the brass melt, forming a homogeneous Cu–Zn–Fe–Ni melt. After EW, the melt rapidly cools down and crystallizes to comprise an ultrafine grained structure with high hardness. Presumably, this crystal structure is a disordered Cu–Zn–Fe–Ni solid solution. Since the EW process is a very fast process, the formation of the Cu–Zn–Fe–Ni phase can be considered adiabatic.

**Figure 8:**
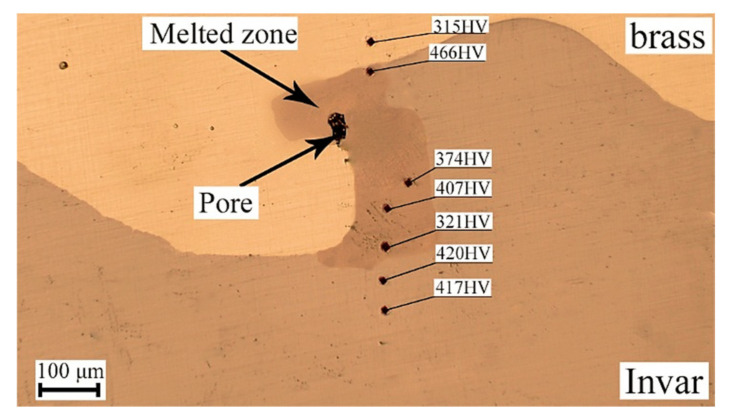
Measurement of microhardness in the melted zone and its vicinity.

Figure 8 shows the positions and results of the microhardness measurements in the melted zone and its vicinity. Invar was harder than brass, as expected. The local values of the microhardness measured within the melted zone considerably varied, from 321 HV to 466 HV. This was obviously due to the heterogeneous microstructure containing differently sized inclusions of Invar.

**Figure 9:**
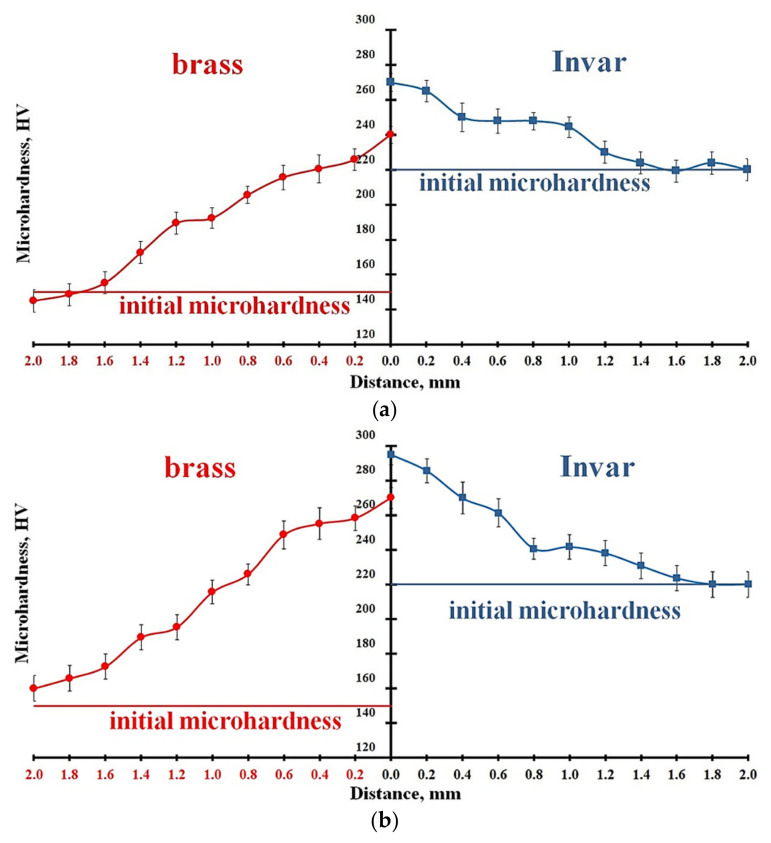
Distribution of microhardness on the weld interface cross-section: (**a**) initial part of bimetal; (**b**) final part of bimetal.

Figure 9 shows the cross-section distribution of microhardness HV in the vicinity of the weld interface. The depth of the strain hardening zone formed during EW was about 1.5 mm on both welded surfaces. The average microhardness of brass near the interface was approximately 40% higher than the microhardness of the initial material. The microhardness of Invar was 20% higher than that of the initial material. The formation of the strain hardening zone is associated with intense collision-induced plastic deformation and substantial differences between the thermophysical parameters of the welded materials. The effect of strain hardening was found to be more pronounced on the brass side. In addition, the microhardness measurements showed that a slight increase in the microhardness values in the final part of the bimetal occurred (Figure 9a,b).

**Figure 10:**
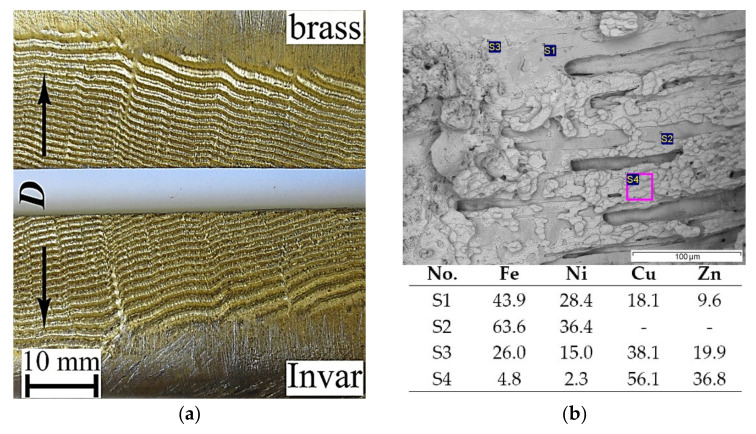
Photograph of the brass and Invar surfaces after the splitting of the bimetal (**a**) and SEM image of Invar surface with point/area EDS analysis results (**b**).

To study the morphology of the brass and Invar surfaces in the final part of the bimetal, it was split into two layers. As illustrated in Figure 10a, both the brass and Invar surfaces exhibited a typical wavy structure with a Cu–Zn–Fe–Ni coating of gold color. Figure 10b shows an SEM image of the Invar surface after splitting. It should be noted that almost the entire surface of Invar was covered with a layer of brass and Cu–Zn–Fe–Ni. The brass surface had a coarse-meshed structure. Presumably, the tearing of brass from Invar likely occurred in the β-phase, because this phase has low plastic properties.

## 4. Conclusions

(1) In the present study, the morphology and crystal structure of a brass–Invar weld interface were investigated. It was shown that the brass–Invar weld interface had a well-defined wavy structure and that the wave parameters increased in the final portion of the bimetal.
(2) The metallographic analysis revealed local melted zones in the crests and troughs of the waves. These melted zones were characterized by a sponge-like porous structure, which was formed due to the dissolution of gases and nonmetallic particles. During the process of solidification, the trapped gases escape from the metal, creating gas-filled pores. Thus, the increase in the wave parameters makes the formation of the melted zones more likely. The melted zones consisted of a disordered Cu–Zn–Fe–Ni solid solution and unmelted Invar particles. Zn and Cu were the main elements in these melted zones.
(3) The observed strain hardening in both brass and Invar indicated their significant plastic flow in the EW process. In this case, the strain hardening of brass was more pronounced and extended to a greater depth than that of Invar. The pattern quality map showed that the high-speed deformation during EW changed the shape of the brass and Invar grains at the weld interface; the grains became strongly elongated in the direction of the most intense metal flow.
(4) Certainly, an increase in the wave amplitude and wavelength leads to an increase in the melted zones at the weld interface. Therefore, it is necessary to choose the explosive welding parameters carefully. Based on the results obtained in the present study, it is proposed to reduce the detonation velocity by 10–15%. Furthermore, it is planned to use the rolling of the explosively welded brass–Invar bimetal to reduce the size of the waves and the destruction of the melted zones.

## Data Availability

Not applicable.
